# Supplementary material for: Real‐time whole‐plant dynamics of heavy metal transport in Arabidopsis halleri and Arabidopsis thaliana by gamma‐ray imaging
Source: Plant Direct. 2019 Apr 23;3(4):e00131. doi: 10.1002/pld3.131 (PMC6589544; doi:10.1002/pld3.131)
Supplement: Supplementary file 2 [file PLD3-3-e00131-s002.docx]

**SUPPLEMENTARY FIGURE LEGENDS**

**Supplementary Figure S1.** Detected gamma-rays from each genotype (columns) and replicate (rows) for root (brown) and shoot (green) ROIs.

**Supplementary Figure S2.** Gamma-rays detected normalized to the 3 h local minima in the root ROI. Pink data points: *A. halleri* wild type. Beige data points: *A. halleri HMA4-RNAi.* Brown data points: *A. thaliana.* Different symbol shapes indicate replicates (*n* = 4 for *A. halleri* wild type and *A. halleri HMA4-*RNAi*,* *n* = 3 for *A. thaliana*).

**SUPPLEMENTARY FIGURES**


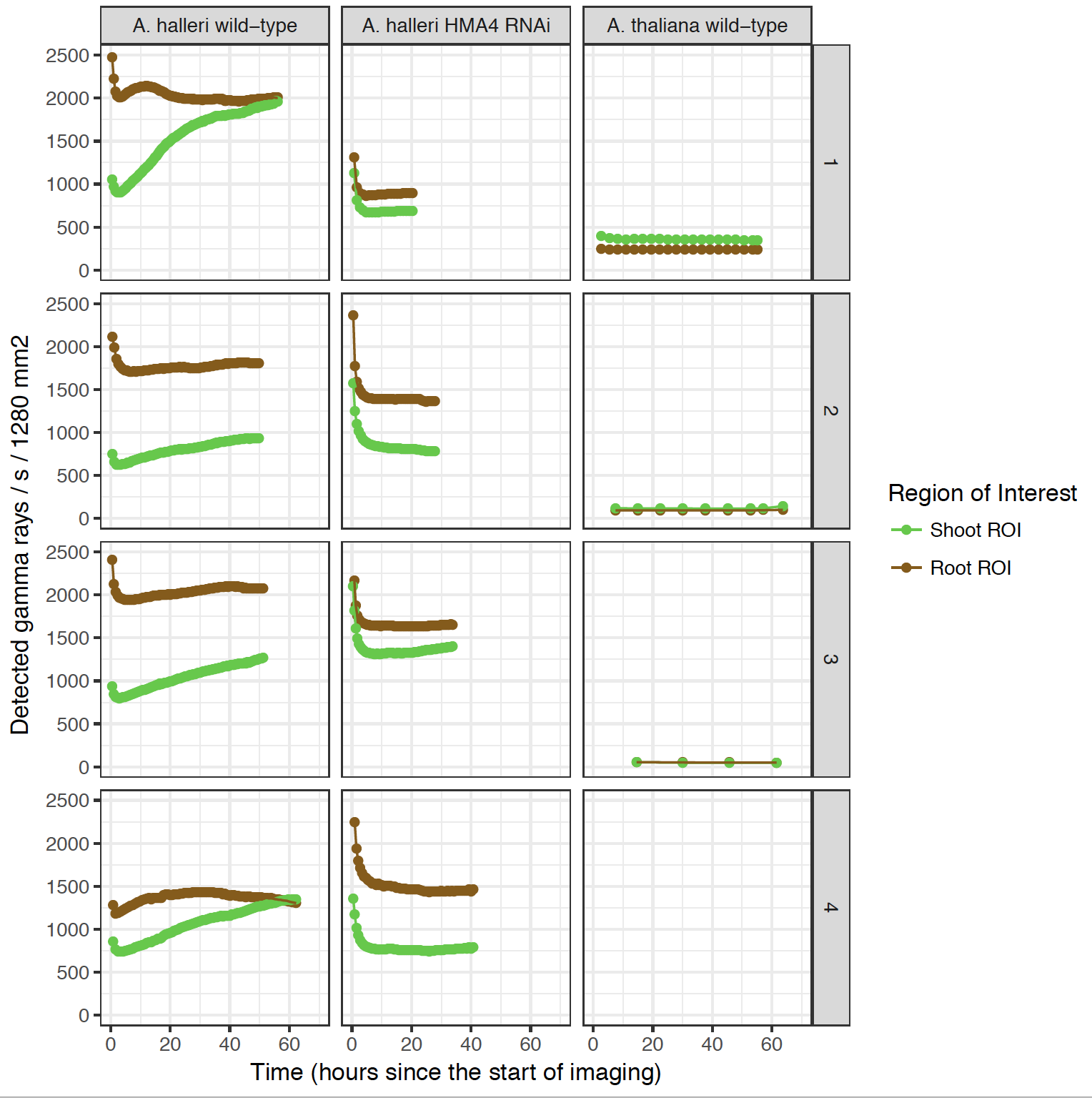


**Supplementary Figure S1.** Detected gamma-rays from each genotype (columns) and replicate (rows) for root (brown) and shoot (green) ROIs.

**Supplementary Figure S2.** Gamma-rays detected normalized to the 3 h local minima in the root ROI. Pink data points: *A. halleri* wild type. Beige data points: *A. halleri HMA4-RNAi.* Brown data points: *A. thaliana.* Different symbol shapes indicate replicates (*n* = 4 for *A. halleri* wild type and *A. halleri HMA4-*RNAi*,* *n* = 3 for *A. thaliana*).
