# Supplementary material for: Real‐time whole‐plant dynamics of heavy metal transport in Arabidopsis halleri and Arabidopsis thaliana by gamma‐ray imaging
Source: Plant Direct. 2019 Apr 23;3(4):e00131. doi: 10.1002/pld3.131 (PMC6589544; doi:10.1002/pld3.131)
Supplement: Supplementary file 1 [file PLD3-3-e00131-s001.docx]

Dear Ivan and Referees,

Thank you for the review of our manuscript, comments and criticisms. We have addressed all comments below. Our responses to the reviewers and editor are in blue font. New text copied from the revised manuscript is indicated in red font. We hope you find the manuscript greatly improved.

All the best,

Siobhan Brady, on behalf of all authors.

**Editor’s Comments**

During an open consultation session between reviewers and editors, it was clear that it is important to demonstrate that the Arabidopsis plants used were actually Zn deficient and if they were, it becomes very relevant to discuss the limitations of the technique (SPECT) in terms of sensitivity and spatial resolution (see reviewer 2 comments). 
Please see our responses to reviewer #2. We have chosen to down-play the statements that these plants were zinc-deprived. We have also directly addressed the comments of reviewer #2 with respect to sensitivity and spatial resolution.

Another minor point is that FRET (page 5, line2) stands for Förster resonance energy transfer, not fluorescence transfer.

This has been edited.

**Reviewer #1:**

1. **Clarify the definition of a time-point and comment on how the unmatched time-points affect the interpretation if any**.

A time point refers to a data file with a time period of 20 minutes to multiple hours. In order to compare time points, interpolated values were calculated. With respect to the question of how the interpolated time points affect interpretation, we posit that they do not at all given the following:

1. Data does not jump widely between the measurements as observed in Figure 2B.
2. Although the data file contains data over a period of time, we also investigated subsetting the data files in order to increase temporal resolution. We found the final data to be reproducible even if subsetted, and thus continued on with the data files with the original periods of time.

We have altered the terminology to better reflect your concerns to “interpolated” time point.

**2. Remove any duplication of methods in the results section.**

We have edited the methods section according to your suggestions below.
 **3. Additionally, I think that the following suggestions would improve the readability and understanding of the manuscript: 
Page1 Line9. Write full genus name in first instance of paper Arabidopsis halleri**

This has been edited.

**4. Page3 Line11. Repeat of information in line10. Suggest to merge sentences for ease of reading.**

We merged the sentences according to your suggestion.

**5. Page5 Line 3-4. Use of commas makes this sentence hard to read.**

This sentence has been edited to improve clarity.

**6. Page5 Line22. Vermiculite / sand ratio?**

3:1 Perlite:sand, edited in the manuscript text accordingly

**7. Page5 Line24. pH of hydroponic solution?**

pH5.8, adjusted with KOH, edited in the manuscript text accordingly

**8. Page5 Line27. A. halleri also grown in vermiculite / sand before cuttings taken?**

*A. halleri* plants were initially grown in Sunshine Mix (3:1 soil:vermiculite). This was edited in the manuscript text.

**9. Page5 Line29. 1x or full strength Sensi-grow?**

This was clarified in the manuscript text: prepared according to the manufacturer’s recommended working concentration

**10. Page6 Line12-13. "12 μmolm-2 s-1 PAR" Check values are correct.**

These values are correct.

**11. Page6 Line15. "to which a small amount" How much?**

200 µCi, edited accordingly in the manuscript text

**12. Page6 Line15-16. Repeat of information in line14. Suggest to merge sentences for ease of reading.**

Sentences have been merged and edited to improve clarity.

**13. Page6. Line18-20. Repeat of information in line16-17. Suggest to merge sentences for ease of reading.**

The sentences have been merged and edited accordingly.

**14. Page8 Line26-28. Time-points not clear. What was calculated using the two adjacent time points?**

An interpolated time value was calculated between the measured time points. This has been edited in the text to improve clarity.

**15. Page10 Line24. This text is more appropriate for discussion section.**

This text has been removed.

**16. Page11 Line6. "including the apoplast" not tested, speculative.**

This has been removed.

**17. Page13 Line14. "respectively" to what?**

This word has been removed.

**18. Fig2B. Units missing.**

This has been edited.

**19. Fig2C. Units missing.**

This has been edited.

**20. Supplementary FigS2. Missing data points? HMA4 rep4 missing in later timepoints.**

The wrong version was uploaded. The new version is correct.

**Reviewer #2**

1. I only have a few concerns. While I think this manuscript ought to be published, I am a bit disappointed by the poor resolution of the imaging. When examining figure 1, only for w.t. A. halleri there is movement of the signal, but with hardly any detail. Could this resolution be improved, or is there a technical limit to the detail that can be discerned by gamma-ray imaging? It is important to discuss this, as it may widen the interest in the techniek.

We have included a section within the discussion that describes the increased sensitivity of the UCD-SPI system relative to other SPECT imaging systems:

Indeed, the UCD-SPI system has extremely high sensitivity (~10%) for a single-photon imaging system, orders of magnitude higher than is found for SPECT systems used in medical imaging (often ~0.01%). This is extremely useful for imaging over long periods of time with high energy gamma rays, as it allows for imaging very low levels of radiotracer and thereby eases the practical issues of radiation safety and shielding and waste generation.

Then, we contrasted the increased sensitivity provided by this system with the tradeoff of poor spatial resolution in a later paragraph within the discussion:

However, there is an inherent tradeoff in increased sensitivity of the UCD-SPI system with spatial resolution. Spatial resolution at the mm-scale could be obtained by using a collimator to better define the spatial origin of detected gamma rays, but this would lead to a greatly reduced event rate in the system. For the high-energy gamma rays of ^65^Zn, collimation is a particular challenge. Given the hours-long time scale of the transport studied here, it is possible that the choice of a collimator could have provide improved event positioning while preserving a usable event rate. A possible hybrid approach could have included using an insertable/removable collimator to acquire an alternating combination of two types of images: high sensitivity-low spatial resolution without the collimator; and low sensitivity-higher spatial resolution with a collimator. However, the use of a collimator for this system is unexplored thus far.

**2. What of course is also a bit disappointing is that in w.t. A. thaliana, there is no sign of Zn uptake. Although authors try to explain this, this is not satisfying. Could it be the A. thaliana plants were not really Zn deficient, so that there is only some absorption of the signal to the root, but no actual uptake (and translocation)?**

First of all, we would like to note that in the previous version of the manuscript **we never referred to *A. thaliana* or *A. halleri* plants as being zinc deficient**. Indeed, the plants of all genotypes were not sick despite pre-growth in low-Zn media, indicating that they are not Zn-limited. Second, Zn uptake rates into the root symplasm **are technically sufficient** for imaging of ^65^Zn fluxes in *A. halleri*, which is why this low-Zn media was used. Physiological deficiency was never the absolute goal of this study. The goal was to determine if the system could be used to **monitor real-time dynamics of Zn flux** in plants, with a genetic control – the HMA4 RNAi *A. halleri* to indicate what happens to Zn flux when HMA4 apoplastic root to shoot transport is defective. We predicted that *A. thaliana* Zn flux would be similar to that of HMA4 RNAi.

In the text we stated that we transferred the plants from sufficient Zn to Zn deprivation media and plants were thus considered as “Zn-deprived”. The concentration of zinc in the media after 19-21 days of growth in “Zn deprivation media” was 0.12 μM and thus the plants were not fully Zn deprived. Thus, this indicates low zinc as opposed to no Zinc.

In order to clarify that we are not studying Zn deficiency , we have adjusted the text as follows:

In the methods: “… without ZnSO4. Zn concentration …” To clarify that no Zn was added to the hydroponic pre-growth medium, it has been changed to: “but omitting ZnSO4. Zn of the medium lacking any added Zn was measured to be 0.12 μM (inductively coupled plasma-atomic emission spectrometry”.

In the methods where it states: “… plant was transferred from the no-Zn Hoagland solution into a solution with radioactive Zn and 1 μM ZnSO4”; it has been changed to: ““… plant was transferred from the low-Zn Hoagland solution into a solution with radioactive Zn and 1 μM ZnSO4”.

In the results where it stated: “…(Hoagland media with no ZnSO4) for hydroponic growth. The plants were allowed to acclimate to Zn deprivation for 19-21 days. ICP-AES analysis of the Zn deprivatio mediat showed that Zn concentration of the media was 0.12uM. ”; it has now been changed to: “(Hoagland media with no added ZnSO4) for hydroponic growth. The plants were allowed to acclimate to Zn deprivation for 19-12 days. ICP-AES analysis of the Zn deprivation media showed that Zn concentration of the media lacking any added Zn was 0.12uM and is thus considered low zinc media”.

All other cases declaring “no zinc”, have been changed to “low zinc”. Furthermore, we have included a statement in the discussion saying that:

**3. Are there data on Zn concentrations before or after a (similar) treatment, to show there is uptake and even translocation? That might clarify this, and such could easily be repeated, without repeating the actual 65Zn application.**

The data in Figure 2D show the concentration of Zn as determined by ICP-AES in the supply media at 0 hours and at 24 hours (the end of the resupply); expressed as a ratio. There is no significant difference between *A. halleri* HMA4 RNAi and *A. thaliana*. There is a net decrease in the amount of available zinc in both *A. halleri* HMA4 RNAi as well as in A. thaliana, although this difference is more variable in *A. thaliana*.

**4. If this will be done, determining expression of some characteristic Zn deficiency induced genes could be done as well, all adding to better understanding the Zn status of particularly the A. thaliana plants and explaining the images.**

While we agree that measuring transcript levels in roots would be nice: 1. We never state that the plants are zinc deficient and instead just that they are grown in Zn deprivation media (which has been changed to “low zinc media). 2. The situation we are monitoring with respect to differences in plant genotype (*halleri.* Vs. *thaliana*) as well as plant size and the existing literature are complex. There is no is no pre-existing absolute marker-based definition of Zn deficiency. Instead, it is always relative. An additional complication of using these marker genes is that they are highly differently expressed between thaliana and halleri. Moreover, in roots, their transcript levels are strongly affected by HMA4-RNAi (as shown for examples of Zn deficiency marker transcripts IRT3 and ZIP4 in Hanikenne et al. (2008)). This makes it somewhat complex to use classical transcriptional markers for Zn deficiency in this specific case.

Therefore, we have chosen not to do these experiments and instead re-iterate in the discussion that there is still a possibility zinc may have not translocated in *A. thaliana*.

**5. I also noticed big differences in plant size between the A. halleri w.t. and the RNAi mutant or the A. thaliana plants. With young plants, it may be more difficult to induce Zn deficiency, which could explain the absence of signal dynamics. This holds especially for A. thaliana, as I appreciate the lack of HMA4 function in the A. halleri RNAi mutant to account for the lack of root-to-shoot transport. Are there any fresh weight or dry weight data to account for these differences?**

We agree that there are large differences in plant size between *A. halleri* and *A. thaliana* plants. We do not have fresh weight or dry weight to account for these differences. We previously stated in the discussion that:

(i) There was no detectable uptake or change of radiolabeled Zn in the shoot
(ii) It is possible that the small size and flat rosette growth habit influenced our ability to detect Zn dynamics
(iii) Low abundance of HMA4 transporters in A. thaliana roots may lead to slower dynamics that we were unable to capture.

Of course, although the net decrease in zinc levels within the resupply media over the 24 hour period suggests that zinc was taken up in *A. thaliana*, with the variability in zinc levels across replicates, we cannot with 100% confidence confirm that zinc was taken up in A. thaliana without an independent method of quantification of 65Zn in the shoot.

We have thus further modified the discussion in the following ways:

(i) “Zn accumulates within the shoot of *A. halleri*, consistent with its ability to hyperaccumulate Zn, different from A. thaliana”

TO

“Zn accumulates within the shoot of *A. halleri*, consistent with its ability to hyperaccumulate Zn, different from the HMA4 RNAi line”

(ii) The speed of Zn transport into the shoot in our data as observed with the smoothed standard error as well as differences with *A. thaliana* show clear shoot accumulation within 5-7 h, respectively

TO

The speed of Zn transport into the shoot in our data as observed with the smoothed standard error show clear shoot accumulation within 5-7 h, respectively

(iii) The paragraph in the discussion describing the A. thaliana results has been changed as follows:

Finally, we compared the dynamics of Zn movement in the Zn hyperaccumulator *A. halleri* with those in the related species *A. thaliana,* a non-metal hyperaccumulator. Based on previous studies comparing Zn-deficient to Zn-sufficient plants of A. thaliana and/or A. halleri, the Zn concentrations in our hydroponic solutions can be estimated to result in moderate Zn deficiency (Talke et al.,2006; Sinclair et al. 2018). The net concentration of ^65^Zn in the resupply media over the 24 hour period showed a net decrease, suggesting that Zn was taken up into the shoot, although these levels are variable. We found that Zn resupply after Zn deprivation in *A. thaliana* did not lead to detectable uptake or change of Zn in the shoot or the root ROI. It is possible that the small size and flat rosette growth habit of *A. thaliana* affected our ability to detect Zn dynamics. Also, low abundance of HMA4 transporters in *A. thaliana* roots may lead to much slower dynamics that we were unable to capture. In the absence of quantification of ^65^Zn levels in the shoot, it is possible, although unlikely, that Zn was not translocated in *A. thaliana*.

**6. Fig2B. Units missing.**

This has been changed in Figure 2B to Figure 2C. The units should be: Average gamma rays detected/s/1280mm^2^
